# Do metal artifact reduction algorithms influence the detection of implant-related injuries to the inferior alveolar canal in CBCT images?

**DOI:** 10.1186/s12903-024-04043-w

**Published:** 2024-02-23

**Authors:** Parisa Soltani, Hugh Devlin, Milad Etemadi Sh, Carlo Rengo, Gianrico Spagnuolo, Kimia Baghaei

**Affiliations:** 1 Department of Oral and Maxillofacial Radiology, Dental Implants Research Center, Dental Research Institute, School of Dentistry, Isfahan University of Medical Sciences, Isfahan, Iran; 2 Department of Neurosciences, Reproductive and Odontostomatological Sciences, University of Naples “Federico II”, Naples, Italy; 3 The Dental School, The University of Bristol, Bristol, UK; 4 Department of Restorative Dentistry, School of Dentistry, Jordan University, Amman, Jordan; 5 Department of Oral and Maxillofacial Surgery, Dental Implants Research Center, Dental Research Institute, School of Dentistry, Isfahan University of Medical Sciences, Isfahan, Iran; 6 Therapeutic Dentistry Department, Institute for Dentistry, Sechenov University, Moscow, 119991 Russia; 7 Student Research Committee, School of Dentistry, Isfahan University of Medical Sciences, Hezar- Jarib Ave, Isfahan, Iran

**Keywords:** Cone beam computed tomography, Dental implant, Inferior alveolar canal, Metal

## Abstract

**Background:**

The routine application of dental implants for replacing missing teeth has revolutionized restorative and prosthetic dentistry. However, cone beam computed tomography (CBCT) evaluations of structures adjacent to the implants are limited by metal artifacts. There are several methods for reducing metal artifacts, but this remains a challenging task. This study aimed to examine the effectiveness of metal artifact reduction (MAR) algorithms in identifying injuries of implants to the inferior alveolar canal in CBCT images.

**Method:**

In this in vitro study, mono-cortical bone windows were created and the inferior alveolar canal was revealed. Using 36 implants, pilot drill and penetration damage of the implant tip into the canal was simulated and compared to the control implants with distance from the canal. CBCT images were evaluated by four experienced observers with and without the MAR algorithm and compared to direct vision as the gold standard. The values of accuracy, sensitivity, and specificity were obtained and compared by receiver operating characteristic (ROC) curve (α = 0.05).

**Result:**

The area under the ROC curve values for detection of pilot drill injuries varied between 0.840–0.917 and 0.639–0.854 in the active and inactive MAR conditions, respectively. The increase in ROC area was only significant for one of the observers (*P* = 0.010). For diagnosing penetrative injuries, the area under the ROC curve values was between 0.990–1.000 and 0.722–1.000 in the active and inactive MAR conditions, respectively. The improvement of ROC curve values in active MAR mode was only significant for one of the observers (*P* = 0.006).

**Conclusion:**

Activation of MAR improved the diagnostic values of CBCT images in detecting both types of implant-related injuries to the inferior alveolar canal. However, for most observers, this increase was not statistically significant.

## Introduction

The routine application of dental implants for replacing missing teeth has revolutionized restorative and prosthetic dentistry. In recent years, there has been an upsurge in demand for dental implants used for prosthetic and aesthetic purposes [1, 2]. Radiology is a frequently employed diagnostic modality in diverse phases of implant therapy encompassing pre-treatment, treatment strategy, implant insertion, and postoperative monitoring [3]. While three-dimensional imaging using cone beam computed tomography (CBCT) is among the most commonly used imaging modalities for the presurgical evaluation and monitoring of symptomatic implants, it is limited by its ability to detect details in areas adjacent to metallic objects or objects with a high atomic number as a result of metal artifacts [4–6].

Although several methods and models have been proposed to reduce these artifacts, the successful elimination of metal-induced artifacts continues to pose a considerable challenge, and the efficacy of the proposed techniques remains a topic of debate [7, 8]. Metal artifact reduction (MAR) algorithms have been developed to repair the gray values altered as a result of artifacts. The effectiveness of MAR algorithms has been studied concerning different metallic or high atomic number materials, from gutta-percha and stainless steel to zirconia, and for different diagnostic tasks, including detection of caries and dental fractures, among other applications [9–11]. The effectiveness of these MAR algorithms has proven to be task-specific [12]. To our knowledge, only one previous research has been found regarding the artifact reduction algorithm’s efficacy in identifying injuries to the inferior alveolar canal, which showed a negative effect of the algorithm [13]. Given the importance of detecting these types of injuries to the inferior alveolar canal and the result being task-specific, this study aimed to examine the effectiveness of the native metal artifact reduction algorithm of Sirona CBCT scanner in identifying injuries of implants to the inferior alveolar canal in CBCT images.

## Methods and materials

The present study was carried out in Department of Oral and Maxillofacial Radiology, Isfahan University of Medical Sciences, Iran. The Research Ethics Committee at Isfahan University of Medical Sciences has approved this study (#IR.MUI.REC.1400.072, approval date: 03/08/2022). Based on the correlation sample size formula the sample size for each group was 12 implants for CI = 95%, α = 0.05 to determine a difference of 0.25 between the control and experimental groups.

### Preparation of specimens

Twelve fresh hemimandibles of sheep (slaughtered on the previous day) were carefully selected, and the soft tissue was completely removed. In order to determine the condition and position of the inferior alveolar canal, an initial radiograph of these hemimandibles was taken with a phosphor plate size 4 (Durr Dental, Bietigheim-Bissingen, Germany). These initial radiographs allowed for screening of the condition of the inferior alveolar canal prior to surgical procedure.

### Surgical procedures and implant insertion

**Fig. 1:**
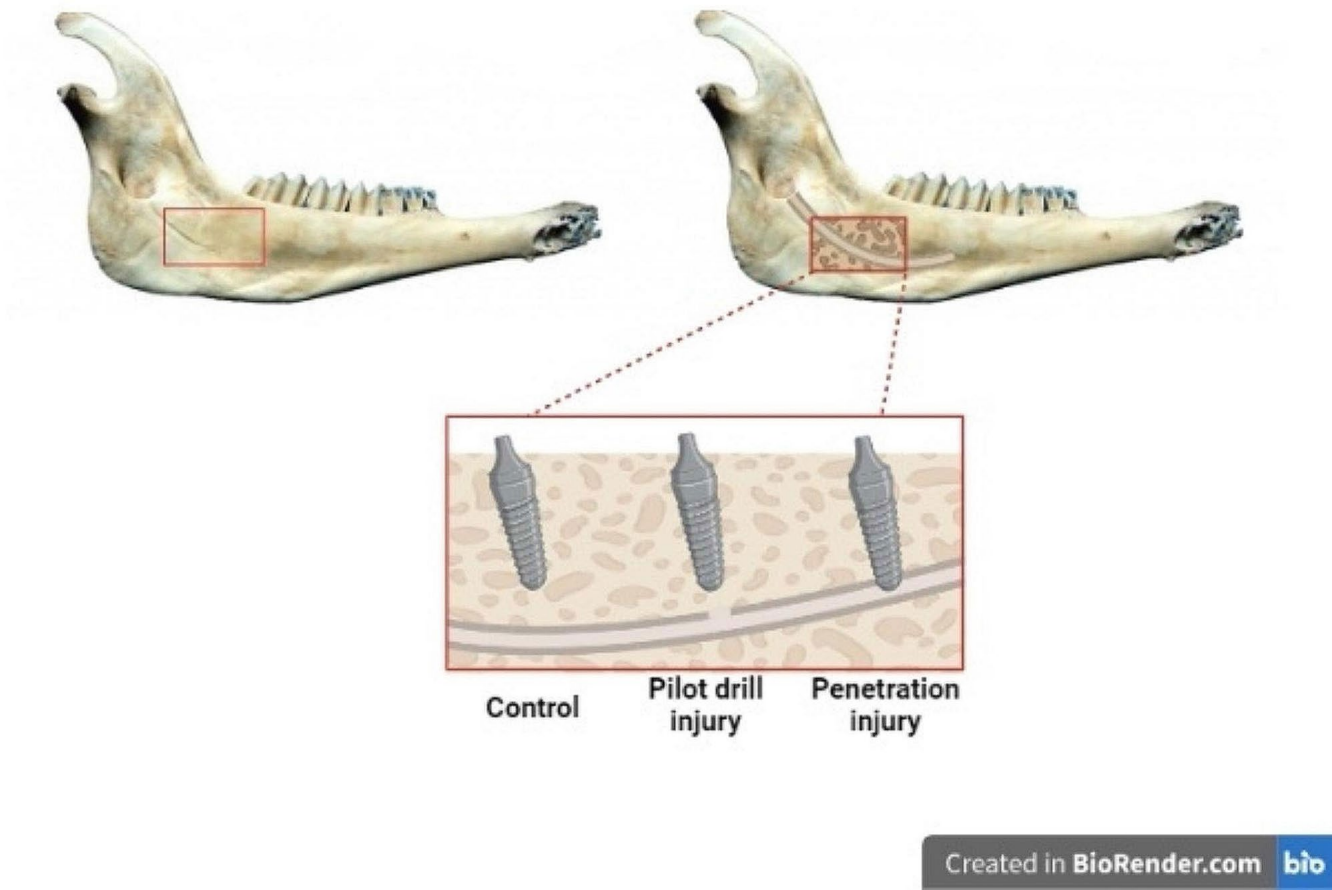
Illustration depicting preparation of sheep mandibles and insertion of dental implants

Monocortical bone windows were created from the lingual side, which allowed for the inferior alveolar canal to be revealed and directly visualized. 36 titanium dental implants (Bionic, Nik Kasht Asia, Tehran, Iran) with sizes 4 × 10 mm and 4 × 12 mm were placed in the mandibles according to the type of injuries. Two types of inferior alveolar nerve injuries were simulated in this study: pilot drill damage and implant tip penetration into the canal. Twelve implants were simulated for each group. In the pilot drill injury, the upper border of the inferior alveolar canal was penetrated by the pilot drill, but the implant tip was placed about 1 mm above the injury site. With implant penetration damage, drilling was done in such a way that the final position of the implant tip was about 1 mm inside the inferior alveolar canal. Twelve implants were placed as a control group 1 mm above the upper border of the canal (Fig. 1). After that, the window was closed again and the hemimandibles were prepared for the imaging stage.

### Preparing the imaging phantom

**Fig. 2:**
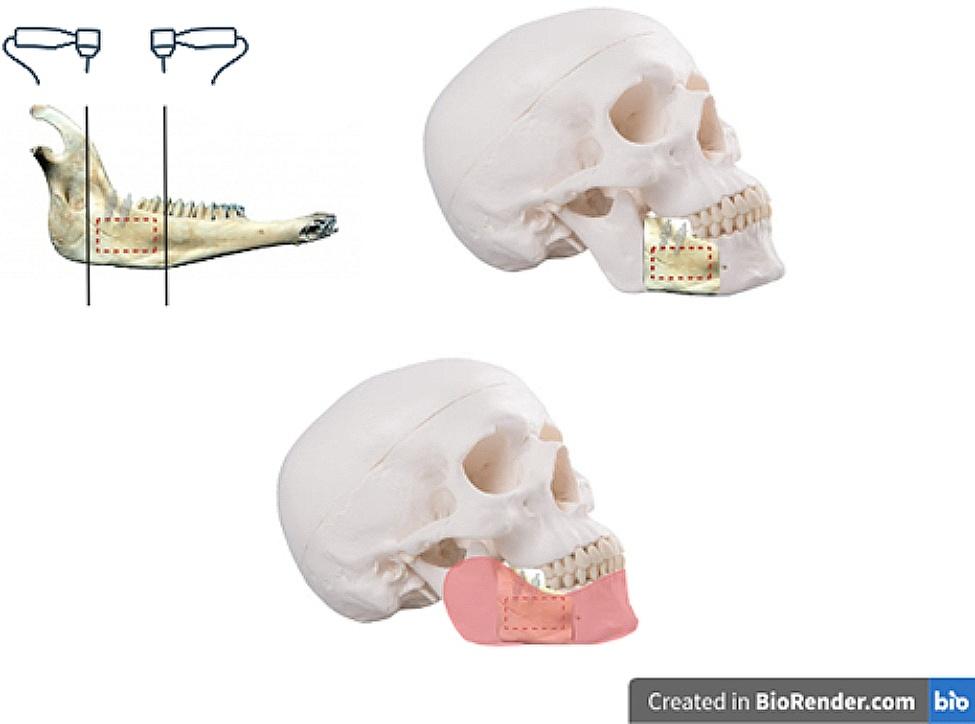
– Illustration depicting preparation of the imaging phantom using cut sheep mandible bone blocks containing implants

The bone segment containing the implants was cut from the hemimandibles using a handpiece. A cranium model (Anatokala, Tehran, Iran) with mandible and maxilla in occlusion was used as an imaging phantom. Bone blocks containing the implants were fixed to the imaging phantom with glue. Soft tissue was replicated by 10 mm of base plate wax (Polywax, Izmir, Turkey) (Fig. 2).

### Preparation of CBCT images

CBCT images were taken by placing the prepared imaging phantom in Galileos scanner (Sirona, Bensheim, Germany) with exposure conditions of 85 kVp, 21 mAs, voxel size 280 micrometers and field of view 15 × 15 cm. For each sample, two scans were performed: with and without activation of MAR algorithm. CBCT images were viewed and evaluated in Sidexis 4 software (Sirona, Bensheim, Germany).

### Observers and reading sessions

**Fig. 3:**
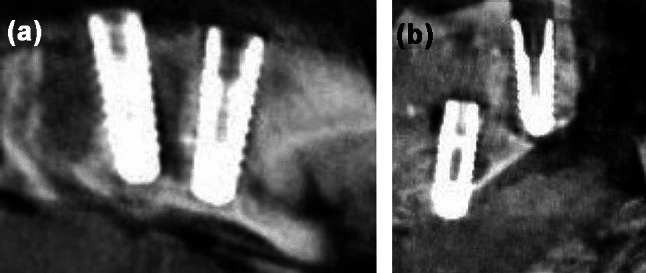
CBCT images of (**a**) control implants (**b**) implants with penetration injury (left on the image) and pilot drill injury (right on the image)

Evaluation of the images was done by 4 observers: one oral and maxillofacial radiologist, two oral and maxillofacial surgeon, and one dentist all with more than 5 years of experience in analysis of CBCT images. A calibration session was held for the observers discussing about three cases unrelated to the study. Evaluation of the images was done in a semi-dark and quiet room. The images in each set (off or on MAR) were provided to each observer on two separate reading sessions with a 2-week interval. The observers were unaware of the presence or absence of damage in the images as well as if the image set they are viewing is with or without the activation of MAR. They also had no contact with each other in the reading sessions. The observers were free to choose the desired views and visual settings for diagnosis (Fig. 3). The observers’ responses were recorded by a 4-point Likert scale: 1 control, 2 pilot drill, 3 implant penetration, 4 uncertain. Uncertain cases were excluded from the analysis.

### Statistical analysis

Statistical analysis was performed using Statistical Package for the Social Sciences (SPSS, version 25, Armonk, NY, USA). Intra- and inter-observer agreements were calculated using Cohen’s kappa. Sensitivity, specificity, and area under the receiver operating characteristic (ROC) curve were calculated for each observer. *P* < 0.05 was considered statistically significant.

## Results

Intraobserver and intraobserver agreements ranged from 0.78 to 0.94 and 0.88 to 0.94, respectively indicating excellent agreements.

**Fig. 4:**
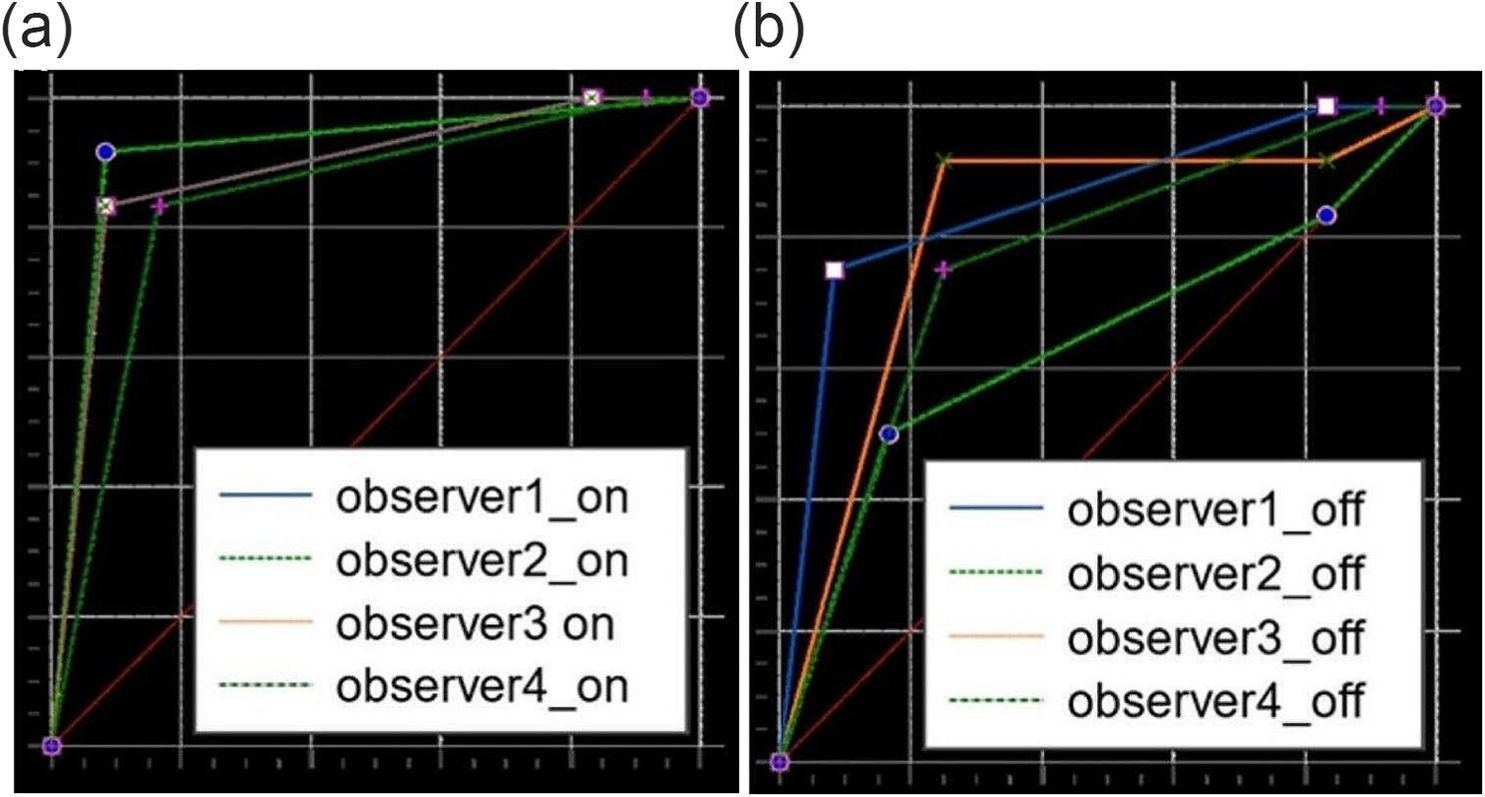
– The receiver operating characteristic (ROC) curves for detection of pilot drill injuries to the inferior alveolar canal with activation of MAR (**a**) and without activation of MAR (**b**)

**Fig. 5:**
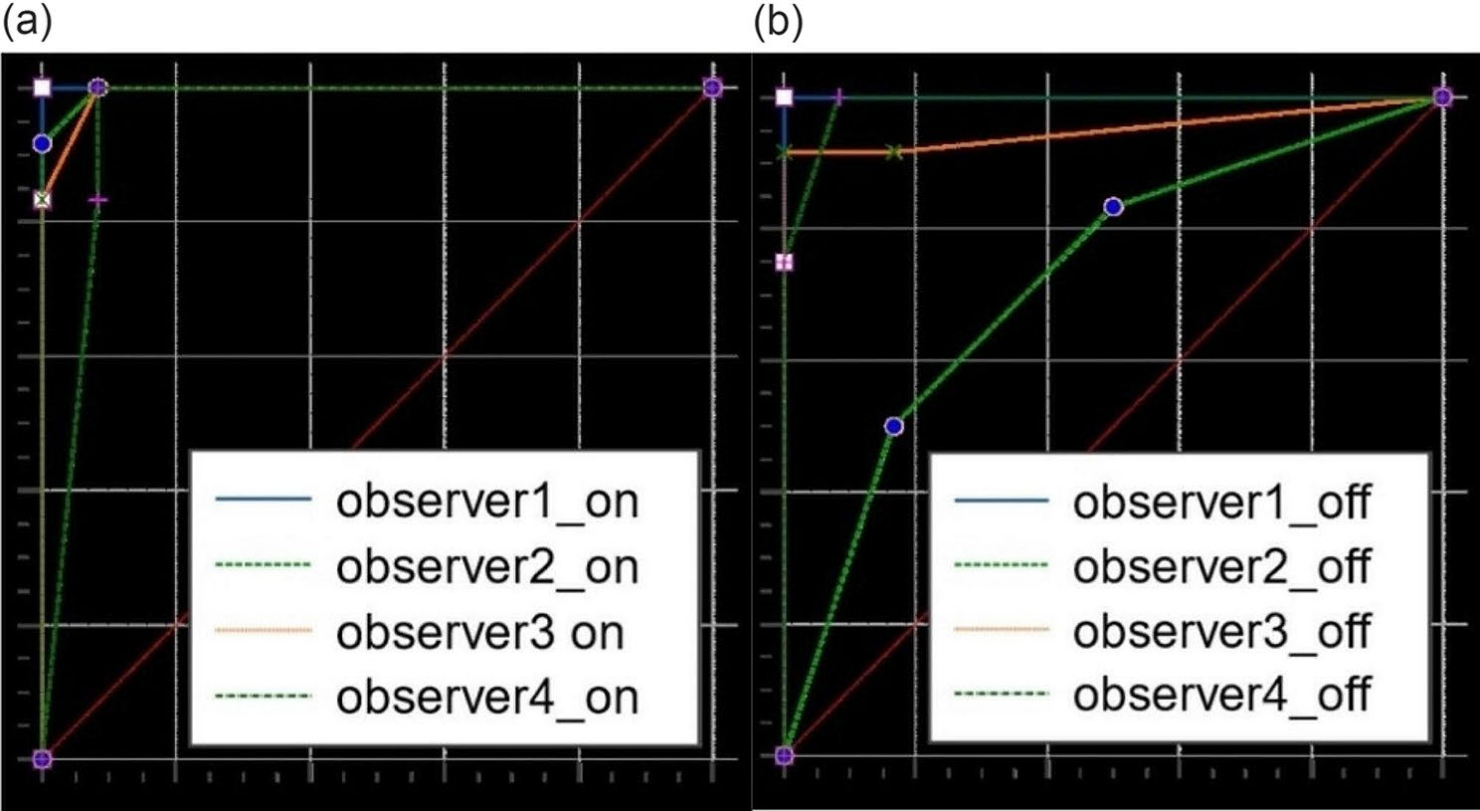
The receiver operating characteristic (ROC) curves for detection of penetrative injuries to the inferior alveolar canal with activation of MAR (**a**) and without activation of MAR (**b**)

Figures 4 and 5 depict the ROC curves of CBCT images with and without activation of MAR for detection of pilot drill and penetrative injuries of the inferior alveolar canal, respectively. The area under ROC curve values for detection of pilot drill injuries ranged between 0.840 and 0.917 in the active MAR and between 0.639 and 0.854 in the inactive MAR conditions. For all observers, activation of MAR led to an increase in the area under the ROC curve when detecting pilot drill injuries to the inferior alveolar canal. However, this difference was not statistically significant (*P* > 0.05), except for one of the observers (*P* = 0.010) (Table 1). For diagnosing penetrative injuries, the area under ROC curve values varied between 0.990 and 1.000 in the active MAR and between 0.722 and 1.000 in the inactive MAR conditions. A similar trend was observed for the detection of penetrative injuries to the inferior alveolar canal, where the area under the ROC curve values was only significantly increased by activation of MAR for one observer (*P* = 0.006) and not significantly different for the other three observers (*P* > 0.05) (Table 2).

**Table 1:** Diagnostic values of CBCT images with/without activation of MAR for detecting pilot drill injuries to the inferior alveolar canal

Observers	MAR condition	AUC	Sensitivity	Specificity	P-value	AUC difference
1	on	0.889	91.67	83.33	0.584	0.035
	off	0.854	91.67	75.00		
2	on	0.917	91.67	91.67	0.010	0.278
	off	0.639	50.00	83.33		
3	on	0.889	83.33	91.67	0.426	0.080
	off	0.809	91.70	75.00		
4	on	0.840	83.33	83.33	0.319	0.080
	off	0.760	75.00	75.00		

MAR: metal artifact reduction

AUC: area under curve

**Table 2:** Diagnostic values of CBCT images with/without activation of MAR for detecting penetrative injuries to the inferior alveolar canal

Observers	MAR condition	AUC	sensitivity	specificity	P value	AUC difference
1	on	1.000	100.00	100.00	0.999<	0.000
	off	1.000	100.00	100.00		
2	on	0.997	91.67	100.00	0.006	0.274
	off	0.722	50.00	83.33		
3	on	0.993	100.00	91.67	0.407	0.042
	off	0.955	100.00	90.91		
4	on	0.990	100.00	91.70	0.749	0.038
	off	0.951	100.00	91.67		

MAR: metal artifact reduction

AUC: area under curve

## Discussion

For detecting implant-related injuries to the inferior alveolar canal, although activation of MAR improved the diagnostic values of CBCT images, this increase was not statistically significant for most observers.

Studies have shown that as high as 13% of implants placed in the posterior mandible can cause injury to the inferior alveolar nerve [14–16]. Mechanical, chemical, and thermal elements can cause injury to the inferior alveolar nerve during or after implant insertion. Direct mechanical trauma from implant drills, implant tip, and bone debris are frequent and can result in pressure on the nerve, its entrapment, or even its rupture [17]. The occurrence of such structural injuries has the potential to give rise to clinical complaints such as anesthesia, hypoesthesia, dysesthesia, or pain. If these injuries are permanent, they affect all aspects of life, reduce the quality of life, and can cause legal problems for dental practitioners [18]. Hence, it is imperative to ensure precise detection of injuries to the inferior alveolar nerve and identify the associated causes and iatrogenic factors.

In this context, CBCT images are helpful in diagnosing complications arising from symptomatic implants. However, as metallic objects with high atomic numbers, these implants produce artifacts including photon starvation, cupping, and beam hardening streaks [19]. The presence of these artifacts can alter CBCT gray values, particularly in the areas immediately adjacent to dental implants, and make the diagnosis of implant complications, such as fenestration, perforation of bony borders, and bone loss challenging. MAR algorithms have been developed to repair these altered gray values. They are usually categorized into five different approaches: the physical modeling-based approach, the projection completion-based approach, the dual energy-based approach, the iterative reconstruction-based approach, and the deep learning-based approach [20].

In the study of de Freitas et al. the influence of metal artifact reduction on the diagnosis of contact between implant and mandibular canal was investigated. They showed that MAR algorithm has a negative effect on the diagnosis. This is in contrast to our findings and may be attributed to the difference in algorithm, the CBCT device, or the difference in implant type [13]. Salemi et al. have investigated the effects of MAR algorithms on the detection of fenestration and dehiscence adjacent to titanium dental implants. Their findings suggested that MAR algorithms did not improve the diagnosis of these bony defects. In fact, accuracy, sensitivity, and specificity decreased in MAR-activated images [21]. The same trend was also observed in the study of Sheikhi et al. in which sensitivity and accuracy were higher in off MAR condition for both fenestration and dehiscence [22]. In the present study, however, the values of area under curve, sensitivity, and specificity generally improved with activation of MAR, although this improvement was not statistically significant for most observers. This inconsistency may be attributed to different diagnostic tasks, as well as different algorithms and CBCT scanners. Salemi et al. used Planmeca Promax 3D and Soredex Cranex 3D CBCT scanners. In the study of Sheikhi et al., similar to our study, Sirona Galileos CBCT unit was used. Moreover, our study applied more observers. In another study, Bagis et al. reported that MAR algorithm improved the accuracy of detection of peri-implant fenestrations. Additionally, MAR algorithm was more effective in enhancing the diagnosis for titanium implants compared to zirconium ones [23]. The implants used in the present study were titanium grade 5 alloys with 6 wt% aluminum and 4% vanadium. The effect of MAR on reducing artifacts arising from different implant materials and alloys is another topic worth further exploring.

Previous studies have reported observer variability for diagnosing implant complications with or without MAR. Salemi et al. noted poor to moderate and good to excellent agreement of two experienced radiologists in on and off MAR modes, respectively [21]. Bagis et al. found moderate agreement without MAR and denoising options and very good agreement with MAR option for their three observers [23]. Therefore, in this study we tried to account for the observer variability by using four observers. Our findings indicated excellent agreement of the observers. Observer variability can be attributed to factors including training and experience [24]. This can also explain why enhancements and modifications in radiologic images can affect the diagnosis of one observer, while having no effect on that of another one.

A limitation of the present study was using sheep hemimandibles in which the bone density as well as the cortication of the inferior alveolar canal borders may be different compared to human mandibles. Sheep mandibles have been used in other studies for placement of dental implants [16, 23]. In the present study, the diagnostic potential of activation of MAR algorithm was tested for detection of two types of simulated injuries to the inferior alveolar canal. These defects were simulated in about 1 mm limit of the canal, as in clinical scenarios, these injuries usually originate from minor miscalculations of available bone height in pre-operative radiographic images. Detection of pilot drill injuries to the inferior alveolar canal can be a more challenging task, as the canal borders are not visible in all cases, especially in the presence of detrimental metallic artifacts of dental implants. The findings of this study, when combined with those of other studies, can be useful for clinicians and radiologists in selecting the most appropriate tools in diagnosis of complications of dental implants. Although in most cases, purchasing the metal artifact reduction algorithm requires additional expenses, even a slight diagnostic advantage can justify its application.

## Conclusion

Activation of MAR improved the diagnostic values of CBCT images in detecting implant-related injuries to the inferior alveolar canal. However, this increase was not statistically significant for most observers.

## Data Availability

The datasets used and/or analyzed during the current study available from the corresponding author on reasonable request.
